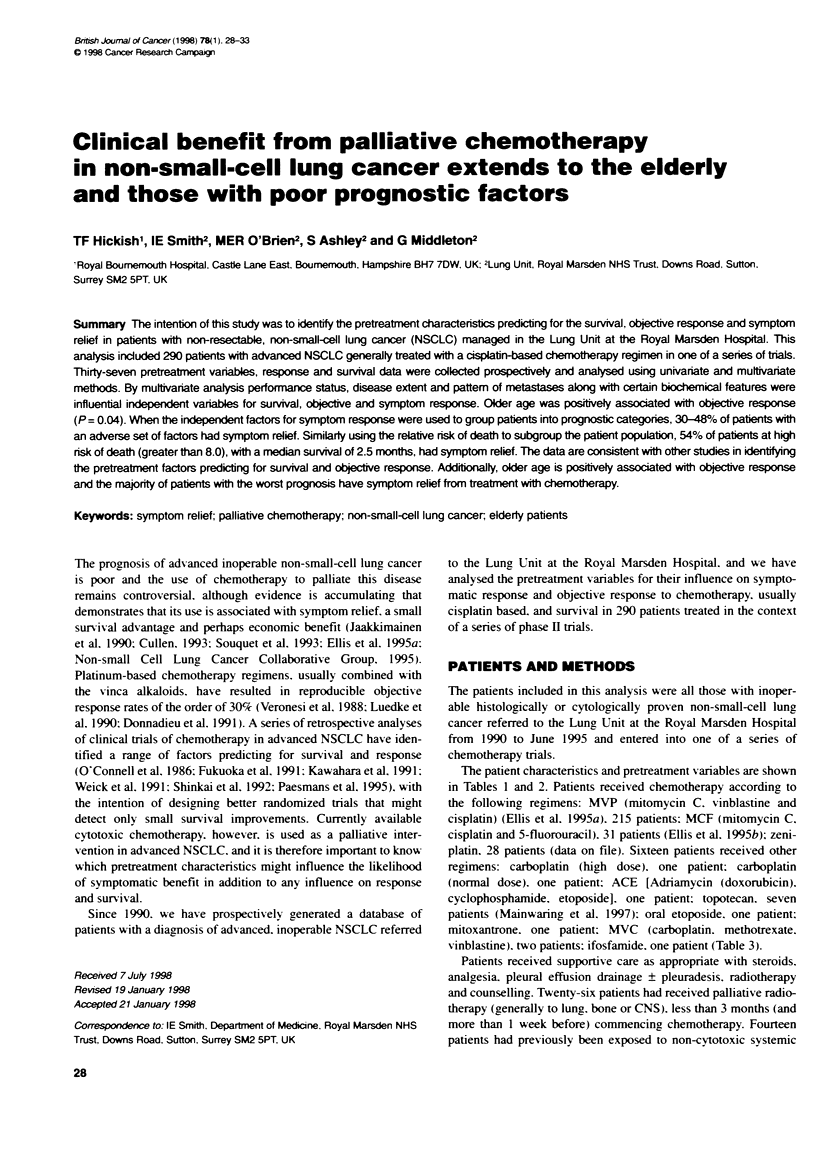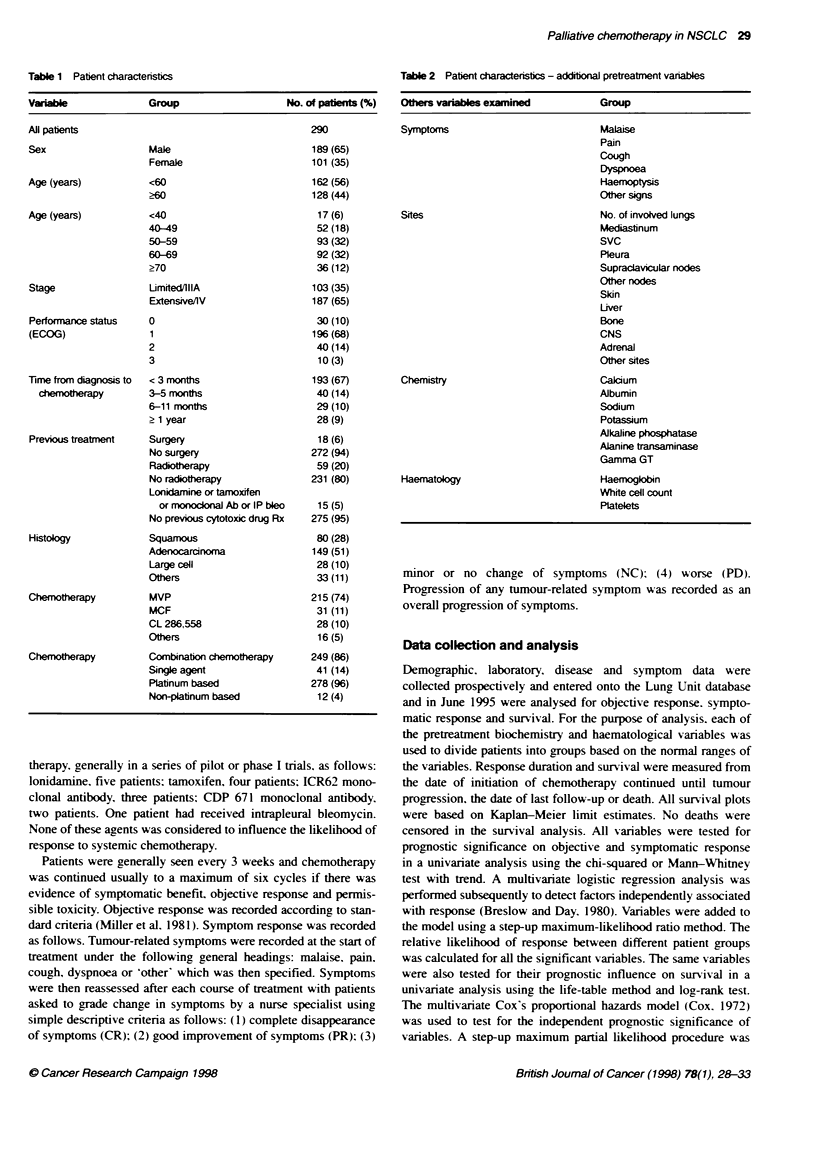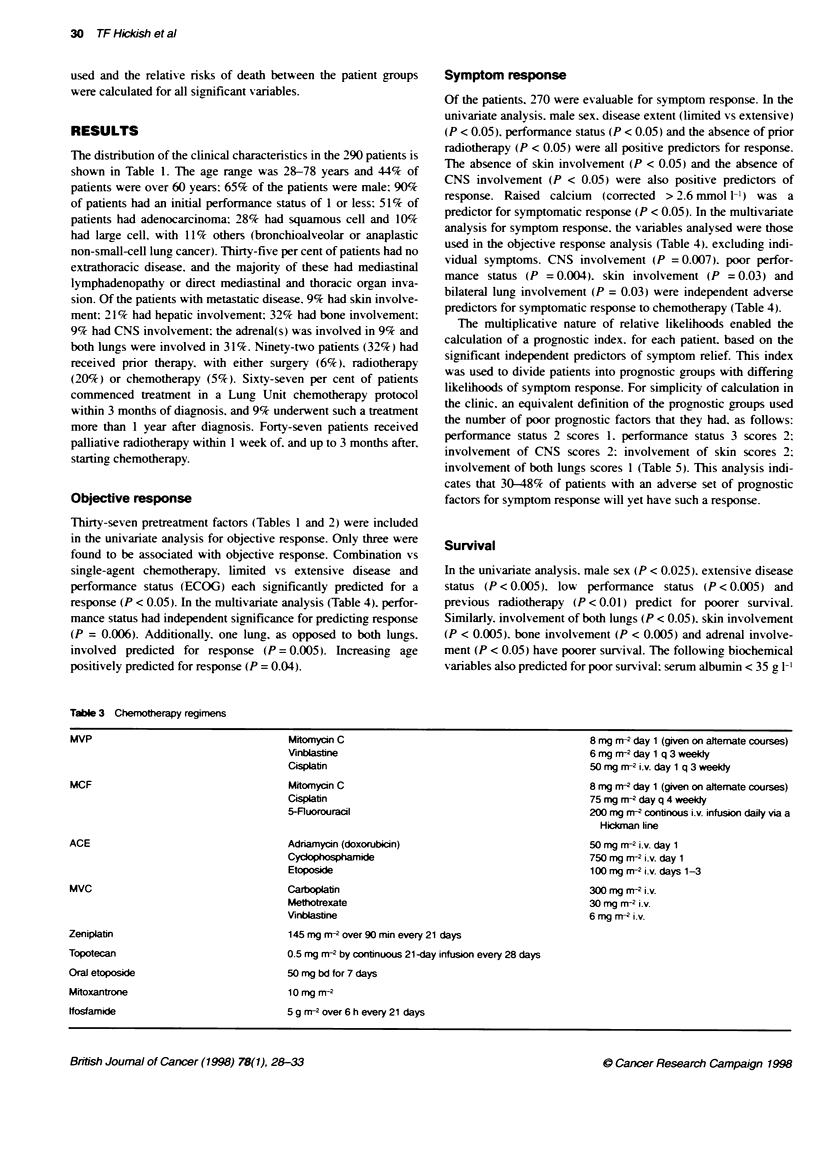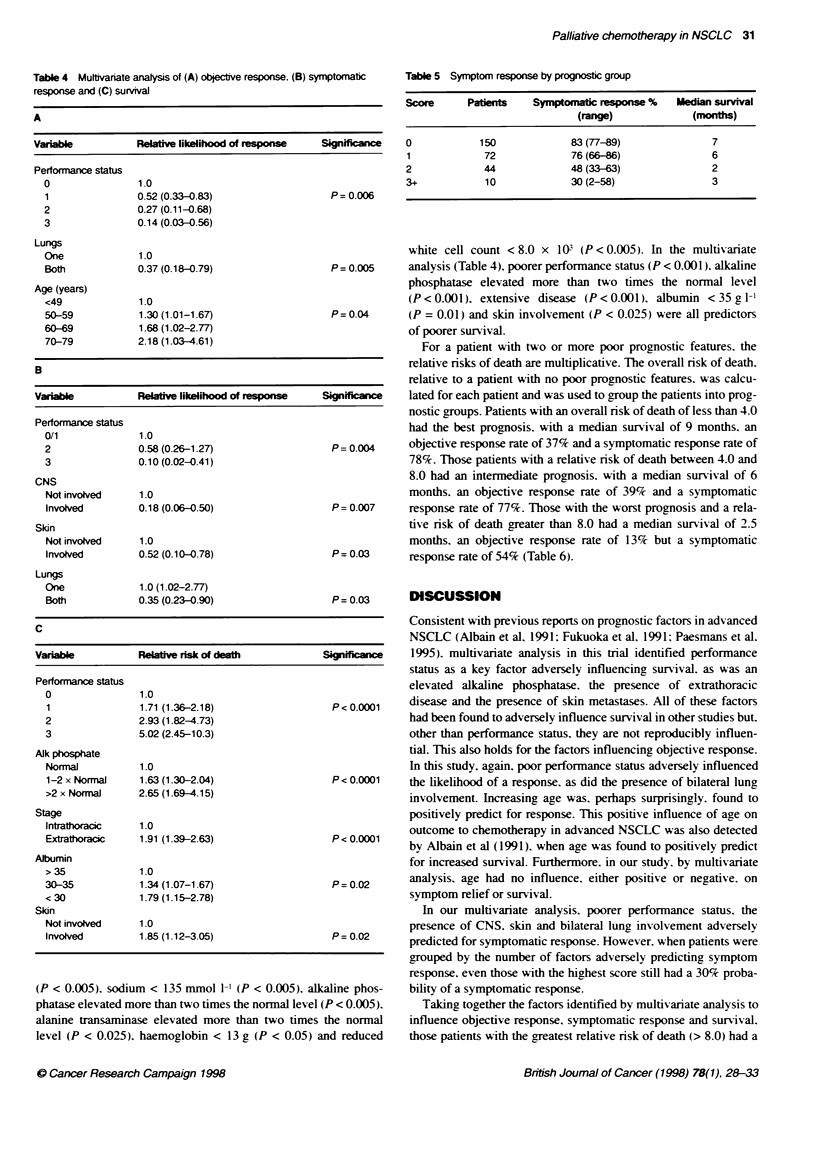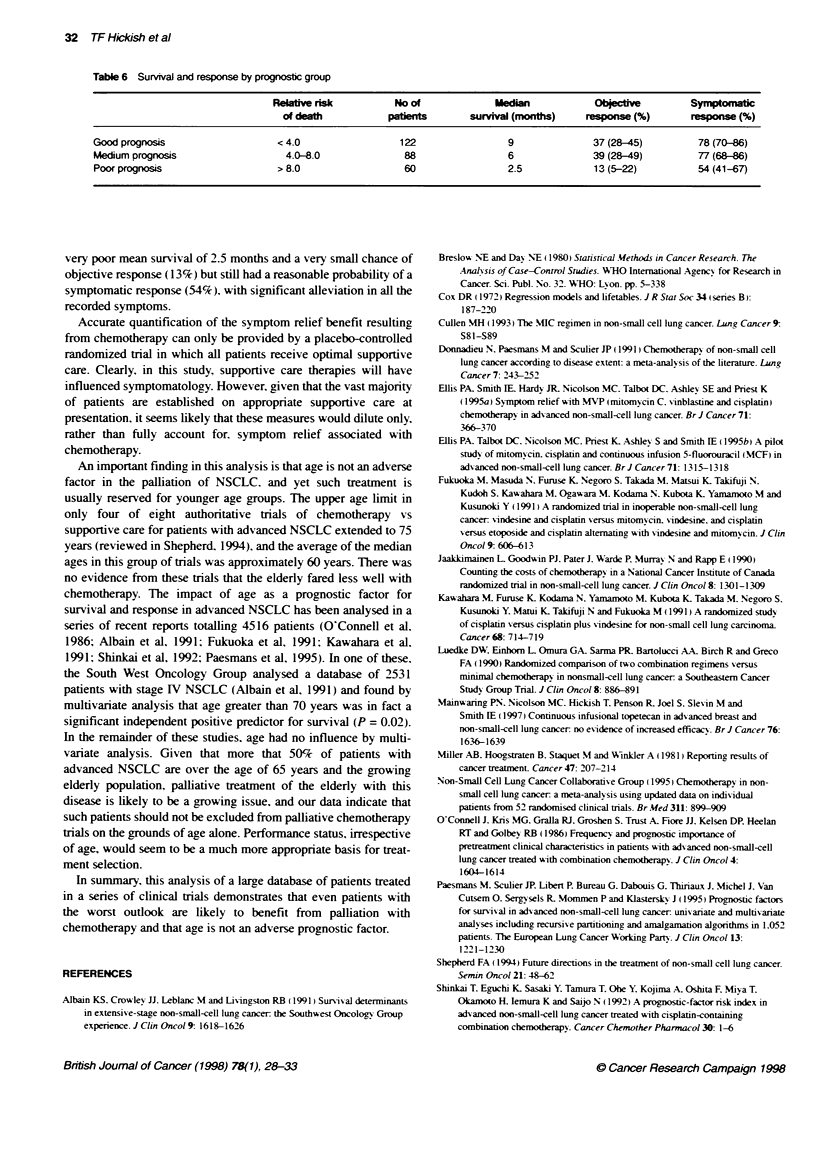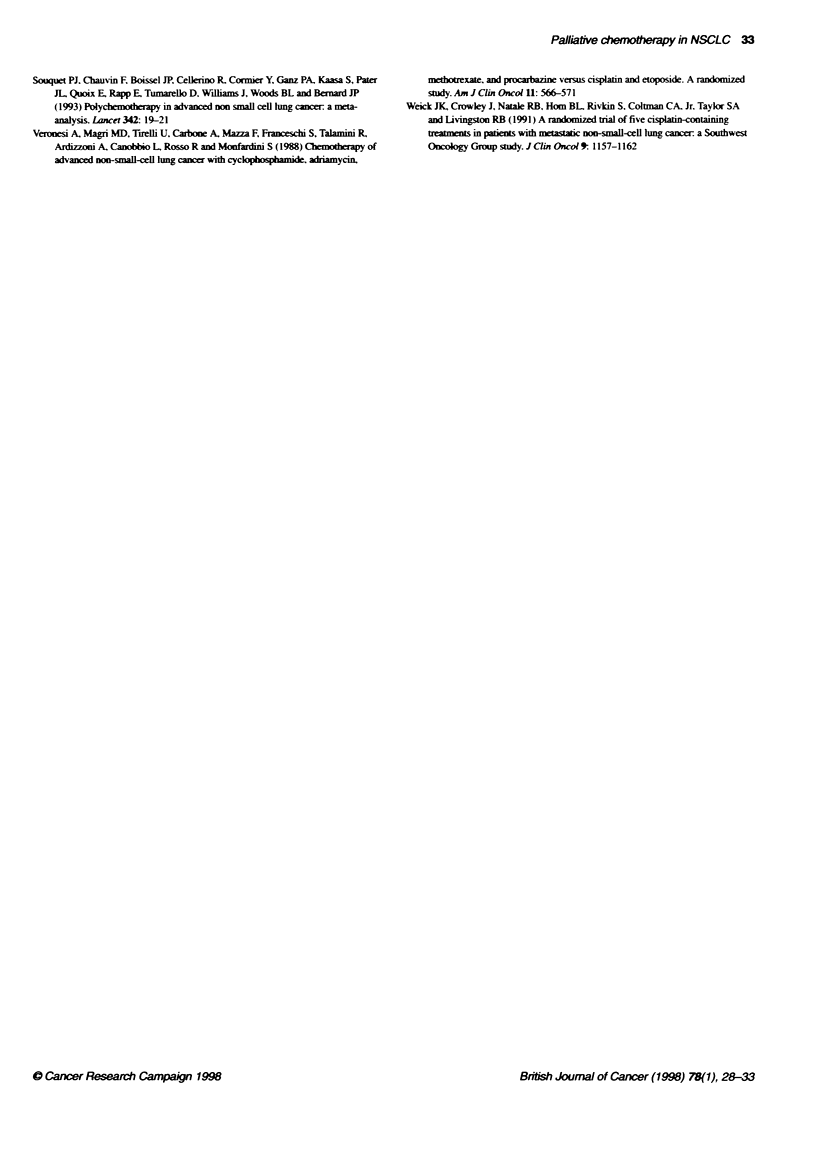# Clinical benefit from palliative chemotherapy in non-small-cell lung cancer extends to the elderly and those with poor prognostic factors.

**DOI:** 10.1038/bjc.1998.437

**Published:** 1998-07

**Authors:** T. F. Hickish, I. E. Smith, M. E. O'Brien, S. Ashley, G. Middleton

**Affiliations:** Royal Bournemouth Hospital, Hampshire, UK.

## Abstract

The intention of this study was to identify the pretreatment characteristics predicting for the survival, objective response and symptom relief in patients with non-resectable, non-small-cell lung cancer (NSCLC) managed in the Lung Unit at the Royal Marsden Hospital. This analysis included 290 patients with advanced NSCLC generally treated with a cisplatin-based chemotherapy regimen in one of a series of trials. Thirty-seven pretreatment variables, response and survival data were collected prospectively and analysed using univariate and multivariate methods. By multivariate analysis performance status, disease extent and pattern of metastases along with certain biochemical features were influential independent variables for survival, objective and symptom response. Older age was positively associated with objective response (P = 0.04). When the independent factors for symptom response were used to group patients into prognostic categories, 30-48% of patients with an adverse set of factors had symptom relief. Similarly using the relative risk of death to subgroup the patient population, 54% of patients at high risk of death (greater than 8.0), with a median survival of 2.5 months, had symptom relief. The data are consistent with other studies in identifying the pretreatment factors predicting for survival and objective response. Additionally, older age is positively associated with objective response and the majority of patients with the worst prognosis have symptom relief from treatment with chemotherapy.


British Journal of Cancer (1998) 78(1). 28–33
© 1998 Cancer Research Campaign

### Clinical benefit from palliative chemotherapy in non-small-cell lung cancer extends to the elderly and those with poor prognostic factors

TF Hickish[1], IE Smith[2], MER O'Brien[2], S Ashley[2] and G Middleton[2]

[1]Royal Bournemouth Hospital, Castle Lane East, Bournemouth, Hampshire BH7 7DW, UK; [2]Lung Unit, Royal Marsden NHS Trust, Downs Road, Sutton, Surrey SM2 5PT, UK

**Summary** The intention of this study was to identify the pretreatment characteristics predicting for the survival, objective response and symptom relief in patients with non-resectable, non-small-cell lung cancer (NSCLC) managed in the Lung Unit at the Royal Marsden Hospital. This analysis included 290 patients with advanced NSCLC generally treated with a cisplatin-based chemotherapy regimen in one of a series of trials. Thirty-seven pretreatment variables, response and survival data were collected prospectively and analysed using univariate and multivariate methods. By multivariate analysis performance status, disease extent and pattern of metastases along with certain biochemical features were influential independent variables for survival, objective and symptom response. Older age was positively associated with objective response ($P = 0.04$). When the independent factors for symptom response were used to group patients into prognostic categories, 30–48% of patients with an adverse set of factors had symptom relief. Similarly using the relative risk of death to subgroup the patient population, 54% of patients at high risk of death (greater than 8.0), with a median survival of 2.5 months, had symptom relief. The data are consistent with other studies in identifying the pretreatment factors predicting for survival and objective response. Additionally, older age is positively associated with objective response and the majority of patients with the worst prognosis have symptom relief from treatment with chemotherapy.

**Keywords:** symptom relief; palliative chemotherapy; non-small-cell lung cancer; elderly patients

The prognosis of advanced inoperable non-small-cell lung cancer is poor and the use of chemotherapy to palliate this disease remains controversial, although evidence is accumulating that demonstrates that its use is associated with symptom relief, a small survival advantage and perhaps economic benefit (Jaakkimainen et al, 1990; Cullen, 1993; Souquet et al, 1993; Ellis et al, 1995*a*; Non-small Cell Lung Cancer Collaborative Group, 1995). Platinum-based chemotherapy regimens, usually combined with the vinca alkaloids, have resulted in reproducible objective response rates of the order of 30% (Veronesi et al, 1988; Luedke et al, 1990; Donnadieu et al, 1991). A series of retrospective analyses of clinical trials of chemotherapy in advanced NSCLC have identified a range of factors predicting for survival and response (O'Connell et al, 1986; Fukuoka et al, 1991; Kawahara et al, 1991; Weick et al, 1991; Shinkai et al, 1992; Paesmans et al, 1995), with the intention of designing better randomized trials that might detect only small survival improvements. Currently available cytotoxic chemotherapy, however, is used as a palliative intervention in advanced NSCLC, and it is therefore important to know which pretreatment characteristics might influence the likelihood of symptomatic benefit in addition to any influence on response and survival.

Since 1990, we have prospectively generated a database of patients with a diagnosis of advanced, inoperable NSCLC referred to the Lung Unit at the Royal Marsden Hospital, and we have analysed the pretreatment variables for their influence on symptomatic response and objective response to chemotherapy, usually cisplatin based, and survival in 290 patients treated in the context of a series of phase II trials.

*Received 7 July 1998*
*Revised 19 January 1998*
*Accepted 21 January 1998*

*Correspondence to:* IE Smith, Department of Medicine, Royal Marsden NHS Trust, Downs Road, Sutton, Surrey SM2 5PT, UK

### PATIENTS AND METHODS

The patients included in this analysis were all those with inoperable histologically or cytologically proven non-small-cell lung cancer referred to the Lung Unit at the Royal Marsden Hospital from 1990 to June 1995 and entered into one of a series of chemotherapy trials.

The patient characteristics and pretreatment variables are shown in Tables 1 and 2. Patients received chemotherapy according to the following regimens: MVP (mitomycin C, vinblastine and cisplatin) (Ellis et al, 1995*a*), 215 patients; MCF (mitomycin C, cisplatin and 5-fluorouracil), 31 patients (Ellis et al, 1995*b*); zeniplatin, 28 patients (data on file). Sixteen patients received other regimens: carboplatin (high dose), one patient; carboplatin (normal dose), one patient; ACE [Adriamycin (doxorubicin), cyclophosphamide, etoposide], one patient; topotecan, seven patients (Mainwaring et al, 1997); oral etoposide, one patient; mitoxantrone, one patient; MVC (carboplatin, methotrexate, vinblastine), two patients; ifosfamide, one patient (Table 3).

Patients received supportive care as appropriate with steroids, analgesia, pleural effusion drainage ± pleuradesis, radiotherapy and counselling. Twenty-six patients had received palliative radiotherapy (generally to lung, bone or CNS), less than 3 months (and more than 1 week before) commencing chemotherapy. Fourteen patients had previously been exposed to non-cytotoxic systemic

**Table 1** Patient characteristics

| Variable | Group | No. of patients (%) |
|---|---|---|
| All patients | | 290 |
| Sex | Male | 189 (65) |
| | Female | 101 (35) |
| Age (years) | <60 | 162 (56) |
| | ≥60 | 128 (44) |
| Age (years) | <40 | 17 (6) |
| | 40–49 | 52 (18) |
| | 50–59 | 93 (32) |
| | 60–69 | 92 (32) |
| | ≥70 | 36 (12) |
| Stage | Limited/IIIA | 103 (35) |
| | Extensive/IV | 187 (65) |
| Performance status (ECOG) | 0 | 30 (10) |
| | 1 | 196 (68) |
| | 2 | 40 (14) |
| | 3 | 10 (3) |
| Time from diagnosis to chemotherapy | < 3 months | 193 (67) |
| | 3–5 months | 40 (14) |
| | 6–11 months | 29 (10) |
| | ≥ 1 year | 28 (9) |
| Previous treatment | Surgery | 18 (6) |
| | No surgery | 272 (94) |
| | Radiotherapy | 59 (20) |
| | No radiotherapy | 231 (80) |
| | Lonidamine or tamoxifen or monoclonal Ab or IP bleo | 15 (5) |
| | No previous cytotoxic drug Rx | 275 (95) |
| Histology | Squamous | 80 (28) |
| | Adenocarcinoma | 149 (51) |
| | Large cell | 28 (10) |
| | Others | 33 (11) |
| Chemotherapy | MVP | 215 (74) |
| | MCF | 31 (11) |
| | CL 286,558 | 28 (10) |
| | Others | 16 (5) |
| Chemotherapy | Combination chemotherapy | 249 (86) |
| | Single agent | 41 (14) |
| | Platinum based | 278 (96) |
| | Non-platinum based | 12 (4) |

therapy, generally in a series of pilot or phase I trials, as follows: lonidamine, five patients; tamoxifen, four patients; ICR62 monoclonal antibody, three patients; CDP 671 monoclonal antibody, two patients. One patient had received intrapleural bleomycin. None of these agents was considered to influence the likelihood of response to systemic chemotherapy.

Patients were generally seen every 3 weeks and chemotherapy was continued usually to a maximum of six cycles if there was evidence of symptomatic benefit, objective response and permissible toxicity. Objective response was recorded according to standard criteria (Miller et al, 1981). Symptom response was recorded as follows. Tumour-related symptoms were recorded at the start of treatment under the following general headings: malaise, pain, cough, dyspnoea or 'other' which was then specified. Symptoms were then reassessed after each course of treatment with patients asked to grade change in symptoms by a nurse specialist using simple descriptive criteria as follows: (1) complete disappearance of symptoms (CR); (2) good improvement of symptoms (PR); (3) minor or no change of symptoms (NC); (4) worse (PD). Progression of any tumour-related symptom was recorded as an overall progression of symptoms.

**Table 2** Patient characteristics – additional pretreatment variables

| Others variables examined | Group |
|---|---|
| Symptoms | Malaise |
| | Pain |
| | Cough |
| | Dyspnoea |
| | Haemoptysis |
| | Other signs |
| Sites | No. of involved lungs |
| | Mediastinum |
| | SVC |
| | Pleura |
| | Supraclavicular nodes |
| | Other nodes |
| | Skin |
| | Liver |
| | Bone |
| | CNS |
| | Adrenal |
| | Other sites |
| Chemistry | Calcium |
| | Albumin |
| | Sodium |
| | Potassium |
| | Alkaline phosphatase |
| | Alanine transaminase |
| | Gamma GT |
| Haematology | Haemoglobin |
| | White cell count |
| | Platelets |

### Data collection and analysis

Demographic, laboratory, disease and symptom data were collected prospectively and entered onto the Lung Unit database and in June 1995 were analysed for objective response, symptomatic response and survival. For the purpose of analysis, each of the pretreatment biochemistry and haematological variables was used to divide patients into groups based on the normal ranges of the variables. Response duration and survival were measured from the date of initiation of chemotherapy continued until tumour progression, the date of last follow-up or death. All survival plots were based on Kaplan–Meier limit estimates. No deaths were censored in the survival analysis. All variables were tested for prognostic significance on objective and symptomatic response in a univariate analysis using the chi-squared or Mann–Whitney test with trend. A multivariate logistic regression analysis was performed subsequently to detect factors independently associated with response (Breslow and Day, 1980). Variables were added to the model using a step-up maximum-likelihood ratio method. The relative likelihood of response between different patient groups was calculated for all the significant variables. The same variables were also tested for their prognostic influence on survival in a univariate analysis using the life-table method and log-rank test. The multivariate Cox's proportional hazards model (Cox, 1972) was used to test for the independent prognostic significance of variables. A step-up maximum partial likelihood procedure was used and the relative risks of death between the patient groups were calculated for all significant variables.

### RESULTS

The distribution of the clinical characteristics in the 290 patients is shown in Table 1. The age range was 28–78 years and 44% of patients were over 60 years; 65% of the patients were male; 90% of patients had an initial performance status of 1 or less; 51% of patients had adenocarcinoma; 28% had squamous cell and 10% had large cell, with 11% others (bronchioalveolar or anaplastic non-small-cell lung cancer). Thirty-five per cent of patients had no extrathoracic disease, and the majority of these had mediastinal lymphadenopathy or direct mediastinal and thoracic organ invasion. Of the patients with metastatic disease, 9% had skin involvement; 21% had hepatic involvement; 32% had bone involvement; 9% had CNS involvement; the adrenal(s) was involved in 9% and both lungs were involved in 31%. Ninety-two patients (32%) had received prior therapy, with either surgery (6%), radiotherapy (20%) or chemotherapy (5%). Sixty-seven per cent of patients commenced treatment in a Lung Unit chemotherapy protocol within 3 months of diagnosis, and 9% underwent such a treatment more than 1 year after diagnosis. Forty-seven patients received palliative radiotherapy within 1 week of, and up to 3 months after, starting chemotherapy.

### Objective response

Thirty-seven pretreatment factors (Tables 1 and 2) were included in the univariate analysis for objective response. Only three were found to be associated with objective response. Combination vs single-agent chemotherapy, limited vs extensive disease and performance status (ECOG) each significantly predicted for a response ($P < 0.05$). In the multivariate analysis (Table 4), performance status had independent significance for predicting response ($P = 0.006$). Additionally, one lung, as opposed to both lungs, involved predicted for response ($P = 0.005$). Increasing age positively predicted for response ($P = 0.04$).

### Symptom response

Of the patients, 270 were evaluable for symptom response. In the univariate analysis, male sex, disease extent (limited vs extensive) ($P < 0.05$), performance status ($P < 0.05$) and the absence of prior radiotherapy ($P < 0.05$) were all positive predictors for response. The absence of skin involvement ($P < 0.05$) and the absence of CNS involvement ($P < 0.05$) were also positive predictors of response. Raised calcium (corrected $> 2.6$ mmol $l^{-1}$) was a predictor for symptomatic response ($P < 0.05$). In the multivariate analysis for symptom response, the variables analysed were those used in the objective response analysis (Table 4), excluding individual symptoms. CNS involvement ($P = 0.007$), poor performance status ($P = 0.004$), skin involvement ($P = 0.03$) and bilateral lung involvement ($P = 0.03$) were independent adverse predictors for symptomatic response to chemotherapy (Table 4).

The multiplicative nature of relative likelihoods enabled the calculation of a prognostic index, for each patient, based on the significant independent predictors of symptom relief. This index was used to divide patients into prognostic groups with differing likelihoods of symptom response. For simplicity of calculation in the clinic, an equivalent definition of the prognostic groups used the number of poor prognostic factors that they had, as follows: performance status 2 scores 1, performance status 3 scores 2; involvement of CNS scores 2; involvement of skin scores 2; involvement of both lungs scores 1 (Table 5). This analysis indicates that 30–48% of patients with an adverse set of prognostic factors for symptom response will yet have such a response.

### Survival

In the univariate analysis, male sex ($P < 0.025$), extensive disease status ($P < 0.005$), low performance status ($P < 0.005$) and previous radiotherapy ($P < 0.01$) predict for poorer survival. Similarly, involvement of both lungs ($P < 0.05$), skin involvement ($P < 0.005$), bone involvement ($P < 0.005$) and adrenal involvement ($P < 0.05$) have poorer survival. The following biochemical variables also predicted for poor survival; serum albumin < 35 g $l^{-1}$

**Table 3** Chemotherapy regimens

| | | |
|---|---|---|
| MVP | Mitomycin C<br>Vinblastine<br>Cisplatin | 8 mg m$^{-2}$ day 1 (given on alternate courses)<br>6 mg m$^{-2}$ day 1 q 3 weekly<br>50 mg m$^{-2}$ i.v. day 1 q 3 weekly |
| MCF | Mitomycin C<br>Cisplatin<br>5-Fluorouracil | 8 mg m$^{-2}$ day 1 (given on alternate courses)<br>75 mg m$^{-2}$ day q 4 weekly<br>200 mg m$^{-2}$ continous i.v. infusion daily via a Hickman line |
| ACE | Adriamycin (doxorubicin)<br>Cyclophosphamide<br>Etoposide | 50 mg m$^{-2}$ i.v. day 1<br>750 mg m$^{-2}$ i.v. day 1<br>100 mg m$^{-2}$ i.v. days 1–3 |
| MVC | Carboplatin<br>Methotrexate<br>Vinblastine | 300 mg m$^{-2}$ i.v.<br>30 mg m$^{-2}$ i.v.<br>6 mg m$^{-2}$ i.v. |
| Zeniplatin | 145 mg m$^{-2}$ over 90 min every 21 days | |
| Topotecan | 0.5 mg m$^{-2}$ by continuous 21-day infusion every 28 days | |
| Oral etoposide | 50 mg bd for 7 days | |
| Mitoxantrone | 10 mg m$^{-2}$ | |
| Ifosfamide | 5 g m$^{-2}$ over 6 h every 21 days | |

© Cancer Research Campaign 1998

**Table 4** Multivariate analysis of (**A**) objective response, (**B**) symptomatic response and (**C**) survival

**A**

| Variable | Relative likelihood of response | Significance |
|---|---|---|
| Performance status | | |
| 0 | 1.0 | |
| 1 | 0.52 (0.33–0.83) | $P = 0.006$ |
| 2 | 0.27 (0.11–0.68) | |
| 3 | 0.14 (0.03–0.56) | |
| Lungs | | |
| One | 1.0 | |
| Both | 0.37 (0.18–0.79) | $P = 0.005$ |
| Age (years) | | |
| <49 | 1.0 | |
| 50–59 | 1.30 (1.01–1.67) | $P = 0.04$ |
| 60–69 | 1.68 (1.02–2.77) | |
| 70–79 | 2.18 (1.03–4.61) | |

**B**

| Variable | Relative likelihood of response | Significance |
|---|---|---|
| Performance status | | |
| 0/1 | 1.0 | |
| 2 | 0.58 (0.26–1.27) | $P = 0.004$ |
| 3 | 0.10 (0.02–0.41) | |
| CNS | | |
| Not involved | 1.0 | |
| Involved | 0.18 (0.06–0.50) | $P = 0.007$ |
| Skin | | |
| Not involved | 1.0 | |
| Involved | 0.52 (0.10–0.78) | $P = 0.03$ |
| Lungs | | |
| One | 1.0 (1.02–2.77) | |
| Both | 0.35 (0.23–0.90) | $P = 0.03$ |

**C**

| Variable | Relative risk of death | Significance |
|---|---|---|
| Performance status | | |
| 0 | 1.0 | |
| 1 | 1.71 (1.36–2.18) | $P < 0.0001$ |
| 2 | 2.93 (1.82–4.73) | |
| 3 | 5.02 (2.45–10.3) | |
| Alk phosphate | | |
| Normal | 1.0 | |
| 1–2 × Normal | 1.63 (1.30–2.04) | $P < 0.0001$ |
| >2 × Normal | 2.65 (1.69–4.15) | |
| Stage | | |
| Intrathoracic | 1.0 | |
| Extrathoracic | 1.91 (1.39–2.63) | $P < 0.0001$ |
| Albumin | | |
| > 35 | 1.0 | |
| 30–35 | 1.34 (1.07–1.67) | $P = 0.02$ |
| < 30 | 1.79 (1.15–2.78) | |
| Skin | | |
| Not involved | 1.0 | |
| Involved | 1.85 (1.12–3.05) | $P = 0.02$ |

($P < 0.005$), sodium < 135 mmol $l^{-1}$ ($P < 0.005$), alkaline phosphatase elevated more than two times the normal level ($P < 0.005$), alanine transaminase elevated more than two times the normal level ($P < 0.025$), haemoglobin < 13 g ($P < 0.05$) and reduced white cell count < 8.0 × $10^3$ ($P < 0.005$). In the multivariate analysis (Table 4), poorer performance status ($P < 0.001$), alkaline phosphatase elevated more than two times the normal level ($P < 0.001$), extensive disease ($P < 0.001$), albumin < 35 g $l^{-1}$ ($P = 0.01$) and skin involvement ($P < 0.025$) were all predictors of poorer survival.

**Table 5** Symptom response by prognostic group

| Score | Patients | Symptomatic response % (range) | Median survival (months) |
|---|---|---|---|
| 0 | 150 | 83 (77–89) | 7 |
| 1 | 72 | 76 (66–86) | 6 |
| 2 | 44 | 48 (33–63) | 2 |
| 3+ | 10 | 30 (2–58) | 3 |

For a patient with two or more poor prognostic features, the relative risks of death are multiplicative. The overall risk of death, relative to a patient with no poor prognostic features, was calculated for each patient and was used to group the patients into prognostic groups. Patients with an overall risk of death of less than 4.0 had the best prognosis, with a median survival of 9 months, an objective response rate of 37% and a symptomatic response rate of 78%. Those patients with a relative risk of death between 4.0 and 8.0 had an intermediate prognosis, with a median survival of 6 months, an objective response rate of 39% and a symptomatic response rate of 77%. Those with the worst prognosis and a relative risk of death greater than 8.0 had a median survival of 2.5 months, an objective response rate of 13% but a symptomatic response rate of 54% (Table 6).

### DISCUSSION

Consistent with previous reports on prognostic factors in advanced NSCLC (Albain et al, 1991; Fukuoka et al, 1991; Paesmans et al, 1995), multivariate analysis in this trial identified performance status as a key factor adversely influencing survival, as was an elevated alkaline phosphatase, the presence of extrathoracic disease and the presence of skin metastases. All of these factors had been found to adversely influence survival in other studies but, other than performance status, they are not reproducibly influential. This also holds for the factors influencing objective response. In this study, again, poor performance status adversely influenced the likelihood of a response, as did the presence of bilateral lung involvement. Increasing age was, perhaps surprisingly, found to positively predict for response. This positive influence of age on outcome to chemotherapy in advanced NSCLC was also detected by Albain et al (1991), when age was found to positively predict for increased survival. Furthermore, in our study, by multivariate analysis, age had no influence, either positive or negative, on symptom relief or survival.

In our multivariate analysis, poorer performance status, the presence of CNS, skin and bilateral lung involvement adversely predicted for symptomatic response. However, when patients were grouped by the number of factors adversely predicting symptom response, even those with the highest score still had a 30% probability of a symptomatic response.

Taking together the factors identified by multivariate analysis to influence objective response, symptomatic response and survival, those patients with the greatest relative risk of death (> 8.0) had a

© Cancer Research Campaign 1998 

**Table 6** Survival and response by prognostic group

| | Relative risk of death | No of patients | Median survival (months) | Objective response (%) | Symptomatic response (%) |
|---|---|---|---|---|---|
| Good prognosis | < 4.0 | 122 | 9 | 37 (28–45) | 78 (70–86) |
| Medium prognosis | 4.0–8.0 | 88 | 6 | 39 (28–49) | 77 (68–86) |
| Poor prognosis | > 8.0 | 60 | 2.5 | 13 (5–22) | 54 (41–67) |

very poor mean survival of 2.5 months and a very small chance of objective response (13%) but still had a reasonable probability of a symptomatic response (54%), with significant alleviation in all the recorded symptoms.

Accurate quantification of the symptom relief benefit resulting from chemotherapy can only be provided by a placebo-controlled randomized trial in which all patients receive optimal supportive care. Clearly, in this study, supportive care therapies will have influenced symptomatology. However, given that the vast majority of patients are established on appropriate supportive care at presentation, it seems likely that these measures would dilute only, rather than fully account for, symptom relief associated with chemotherapy.

An important finding in this analysis is that age is not an adverse factor in the palliation of NSCLC, and yet such treatment is usually reserved for younger age groups. The upper age limit in only four of eight authoritative trials of chemotherapy vs supportive care for patients with advanced NSCLC extended to 75 years (reviewed in Shepherd, 1994), and the average of the median ages in this group of trials was approximately 60 years. There was no evidence from these trials that the elderly fared less well with chemotherapy. The impact of age as a prognostic factor for survival and response in advanced NSCLC has been analysed in a series of recent reports totalling 4516 patients (O'Connell et al, 1986; Albain et al, 1991; Fukuoka et al, 1991; Kawahara et al, 1991; Shinkai et al, 1992; Paesmans et al, 1995). In one of these, the South West Oncology Group analysed a database of 2531 patients with stage IV NSCLC (Albain et al, 1991) and found by multivariate analysis that age greater than 70 years was in fact a significant independent positive predictor for survival ($P = 0.02$). In the remainder of these studies, age had no influence by multivariate analysis. Given that more that 50% of patients with advanced NSCLC are over the age of 65 years and the growing elderly population, palliative treatment of the elderly with this disease is likely to be a growing issue, and our data indicate that such patients should not be excluded from palliative chemotherapy trials on the grounds of age alone. Performance status, irrespective of age, would seem to be a much more appropriate basis for treatment selection.

In summary, this analysis of a large database of patients treated in a series of clinical trials demonstrates that even patients with the worst outlook are likely to benefit from palliation with chemotherapy and that age is not an adverse prognostic factor.

### REFERENCES

Albain KS, Crowley JJ, Leblanc M and Livingston RB (1991) Survival determinants in extensive-stage non-small-cell lung cancer: the Southwest Oncology Group experience. *J Clin Oncol* **9**: 1618–1626

Breslow NE and Day NE (1980) *Statistical Methods in Cancer Research. The Analysis of Case–Control Studies*. WHO International Agency for Research in Cancer. Sci. Publ. No. 32. WHO: Lyon. pp. 5–338

Cox DR (1972) Regression models and lifetables. *J R Stat Soc* **34** (series B): 187–220

Cullen MH (1993) The MIC regimen in non-small cell lung cancer. *Lung Cancer* **9**: S81–S89

Donnadieu N, Paesmans M and Sculier JP (1991) Chemotherapy of non-small cell lung cancer according to disease extent: a meta-analysis of the literature. *Lung Cancer* **7**: 243–252

Ellis PA, Smith IE, Hardy JR, Nicolson MC, Talbot DC, Ashley SE and Priest K (1995*a*) Symptom relief with MVP (mitomycin C, vinblastine and cisplatin) chemotherapy in advanced non-small-cell lung cancer. *Br J Cancer* **71**: 366–370

Ellis PA, Talbot DC, Nicolson MC, Priest K, Ashley S and Smith IE (1995*b*) A pilot study of mitomycin, cisplatin and continuous infusion 5-fluorouracil (MCF) in advanced non-small-cell lung cancer. *Br J Cancer* **71**: 1315–1318

Fukuoka M, Masuda N, Furuse K, Negoro S, Takada M, Matsui K, Takifuji N, Kudoh S, Kawahara M, Ogawara M, Kodama N, Kubota K, Yamamoto M and Kusunoki Y (1991) A randomized trial in inoperable non-small-cell lung cancer: vindesine and cisplatin versus mitomycin, vindesine, and cisplatin versus etoposide and cisplatin alternating with vindesine and mitomycin. *J Clin Oncol* **9**: 606–613

Jaakkimainen L, Goodwin PJ, Pater J, Warde P, Murray N and Rapp E (1990) Counting the costs of chemotherapy in a National Cancer Institute of Canada randomized trial in non-small-cell lung cancer. *J Clin Oncol* **8**: 1301–1309

Kawahara M, Furuse K, Kodama N, Yamamoto M, Kubota K, Takada M, Negoro S, Kusunoki Y, Matui K, Takifuji N and Fukuoka M (1991) A randomized study of cisplatin versus cisplatin plus vindesine for non-small cell lung carcinoma. *Cancer* **68**: 714–719

Luedke DW, Einhorn L, Omura GA, Sarma PR, Bartolucci AA, Birch R and Greco FA (1990) Randomized comparison of two combination regimens versus minimal chemotherapy in nonsmall-cell lung cancer: a Southeastern Cancer Study Group Trial. *J Clin Oncol* **8**: 886–891

Mainwaring PN, Nicolson MC, Hickish T, Penson R, Joel S, Slevin M and Smith IE (1997) Continuous infusional topetecan in advanced breast and non-small-cell lung cancer: no evidence of increased efficacy. *Br J Cancer* **76**: 1636–1639

Miller AB, Hoogstraten B, Staquet M and Winkler A (1981) Reporting results of cancer treatment. *Cancer* **47**: 207–214

Non-Small Cell Lung Cancer Collaborative Group (1995) Chemotherapy in non-small cell lung cancer: a meta-analysis using updated data on individual patients from 52 randomised clinical trials. *Br Med* **311**: 899–909

O'Connell J, Kris MG, Gralla RJ, Groshen S, Trust A, Fiore JJ, Kelsen DP, Heelan RT and Golbey RB (1986) Frequency and prognostic importance of pretreatment clinical characteristics in patients with advanced non-small-cell lung cancer treated with combination chemotherapy. *J Clin Oncol* **4**: 1604–1614

Paesmans M, Sculier JP, Libert P, Bureau G, Dabouis G, Thiriaux J, Michel J, Van Cutsem O, Sergysels R, Mommen P and Klastersky J (1995) Prognostic factors for survival in advanced non-small-cell lung cancer: univariate and multivariate analyses including recursive partitioning and amalgamation algorithms in 1,052 patients. The European Lung Cancer Working Party. *J Clin Oncol* **13**: 1221–1230

Shepherd FA (1994) Future directions in the treatment of non-small cell lung cancer. *Semin Oncol* **21**: 48–62

Shinkai T, Eguchi K, Sasaki Y, Tamura T, Ohe Y, Kojima A, Oshita F, Miya T, Okamoto H, Iemura K and Saijo N (1992) A prognostic-factor risk index in advanced non-small-cell lung cancer treated with cisplatin-containing combination chemotherapy. *Cancer Chemother Pharmacol* **30**: 1–6

© Cancer Research Campaign 1998

Souquet PJ, Chauvin F, Boissel JP, Cellerino R, Cormier Y, Ganz PA, Kaasa S, Pater JL, Quoix E, Rapp E, Tumarello D, Williams J, Woods BL and Bernard JP (1993) Polychemotherapy in advanced non small cell lung cancer: a meta-analysis. *Lancet* **342**: 19–21

Veronesi A, Magri MD, Tirelli U, Carbone A, Mazza F, Franceschi S, Talamini R, Ardizzoni A, Canobbio L, Rosso R and Monfardini S (1988) Chemotherapy of advanced non-small-cell lung cancer with cyclophosphamide, adriamycin, methotrexate, and procarbazine versus cisplatin and etoposide. A randomized study. *Am J Clin Oncol* **11**: 566–571

Weick JK, Crowley J, Natale RB, Hom BL, Rivkin S, Coltman CA, Jr, Taylor SA and Livingston RB (1991) A randomized trial of five cisplatin-containing treatments in patients with metastatic non-small-cell lung cancer: a Southwest Oncology Group study. *J Clin Oncol* **9**: 1157–1162

© *Cancer Research Campaign 1998*